# Endocytosis Regulates Cell Soma Translocation and the Distribution of Adhesion Proteins in Migrating Neurons

**DOI:** 10.1371/journal.pone.0017802

**Published:** 2011-03-22

**Authors:** Jennifer C. Shieh, Bruce T. Schaar, Karpagam Srinivasan, Frances M. Brodsky, Susan K. McConnell

**Affiliations:** 1 Department of Biology, Stanford University, Stanford, California, United States of America; 2 Program in Neuroscience, Stanford University, Stanford, California, United States of America; 3 Department of Developmental Biology, Stanford University, Stanford, California, United States of America; 4 Departments of Bioengineering and Therapeutic Sciences, Pharmaceutical Chemistry, and Microbiology and Immunology, University of California San Francisco, San Francisco, California, United States of America; University of Birmingham, United Kingdom

## Abstract

Newborn neurons migrate from their birthplace to their final location to form a properly functioning nervous system. During these movements, young neurons must attach and subsequently detach from their substrate to facilitate migration, but little is known about the mechanisms cells use to release their attachments. We show that the machinery for clathrin-mediated endocytosis is positioned to regulate the distribution of adhesion proteins in a subcellular region just proximal to the neuronal cell body. Inhibiting clathrin or dynamin function impedes the movement of migrating neurons both *in vitro* and *in vivo*. Inhibiting dynamin function *in vitro* shifts the distribution of adhesion proteins to the rear of the cell. These results suggest that endocytosis may play a critical role in regulating substrate detachment to enable cell body translocation in migrating neurons.

## Introduction

Neuronal migration is critical for nervous system development. Disruptions in migration have been implicated in neurological disorders such as epilepsy, mental retardation, schizophrenia, and dyslexia [1], [2], [3]. Many of these disruptions are linked to cytoskeletal dysregulation, which impairs the directed motility of migrating neurons and prevents them from reaching their final destination. Neurons migrate in a stereotypical saltatory, two-stroke cycle: first, a leading process extends and explores, then the cell soma follows in a distinct translocation event [4], [5], [6]. After the leading process extends but before the cell soma moves forward, a cytoplasmic dilation swells in the leading process proximal to the nucleus [5]. This dilation is characteristic of neurons migrating on many substrates from radial glia to extracellular matrix (ECM) [7], [8], [9], [10]. After dilation formation, the nucleus moves forward into this transient structure, due in part to myosin contractions at the cell rear [5], [11], [12]. The cycle repeats as the neuron propels itself forward.

Neuron migration can also be described by the distinct yet integrated steps of classic cell migration models: polarization and protrusion, attachment at the cell front, forward movement of the cell body, and detachment with retraction at the cell rear [13], [14], [15], [16]. Great progress has been made in elucidating the mechanisms that control these steps in fibroblast-like cells migrating in two dimensions (2D), illuminating the critical roles of Rho GTPases and actin regulation in polarization, protrusion, and translocation [13], [17], [18], [19]. Actin polymerization is also tightly linked to the formation of adhesions at leading edge lamellipodia [20], [21], where macromolecular adhesion complexes surrounding integrin receptors connect ECM molecules like fibronectin or laminin to the actin cytoskeleton. The formation and assembly of adhesion complexes has been studied intensively [15], but less is known about adhesion complex disassembly.

Adhesion disassembly is as important as assembly; the cycle of attachment at the leading edge and detachment at the rear must be properly regulated for forward movement to occur [15]. Stronger adhesions at the leading edge exert tractional forces, while weaker adhesions at the rear enable the cell body to release from the substrate. Altering the balance of adhesion affects migratory speed and indeed whether a cell moves at all [22]. Overly weak adhesions fail to provide sufficient traction for forward movement; conversely, overly strong adhesions cause cells to stick and fail to detach from substrates.

Adhesions can be disassembled through biochemical and mechanical mechanisms [23]. For example, calpain can proteolyze talin binding domains that link integrin receptors to the actin cytoskeleton, thus promoting adhesion disassembly [24]. Biomechanical mechanisms can also drive de-adhesion. In migrating fibroblasts, myosin-based contractions break off pieces of membrane, leaving a trail of membrane footprints on the substrate [25], [26]. Alternatively, endocytic internalization of adhesion molecules can physically disrupt contacts between an ECM substrate and cell membrane. Growing evidence suggests that clathrin-mediated endocytosis (CME) is involved in adhesion disassembly. Disrupting clathrin- or dynamin-dependent endocytosis in fibroblasts or fibrosarcoma cells leads to persistent, large focal adhesions that prevent normal migration [27], [28], [29]. In neurons, growth cone motility and axon elongation require endocytosis of L1 cell adhesion molecules for de-adhesion [30], [31]. L1 interacts with the clathrin adaptor AP-2 and is internalized through CME in axonal growth cones [32]. A neuron-specific L1 isoform potentiates migration in non-neuronal cells by interacting with β1 integrins in a clathrin- and dynamin-dependent manner [33], [34]. Another link between adhesion and endocytosis was recently shown in neurons; disruption of Rab GTPases involved in endocytic trafficking alters N-cadherin distribution [35].

Both histological and functional evidence point to a role for endocytosis in migrating neurons. Electron micrographs show clathrin coated vesicles (CCVs) situated near adhesive contact points in cerebellar granule neurons migrating along radial glia [36], [37] and near adherens junctions in neurons migrating in chains toward the olfactory bulb [38]. Several genes critical for cortical neuron migration are associated with endocytosis and adhesion. RNAi-mediated knock-down of the dyslexia-associated protein KIAA0319 disrupts radial migration; KIAA0319 is a putative adhesion protein that interacts with AP-2 and follows a CME pathway [39], [40]. Disruption of Disabled-1 (Dab1), a protein related to the clathrin adaptor Disabled-2 (Dab2), leads to improper cortical migration and the failure of migrating neurons to detach from radial glia [41]. Dab1 acts via the Reelin pathway to regulate radial migration, potentially by binding to clathrin adaptors such as AP-2 and affecting the internalization and recycling of the Reelin receptors VLDLR and APOE-R2 [42].

Here we study the role of endocytosis in regulating the subcellular distribution of adhesion proteins in migrating neurons and test whether disrupting clathrin or dynamin function alters migration. Ultrastructural examination of neurons migrating *in vitro* reveals that endocytic CCVs are present in the cytoplasmic dilation at points of contact with an ECM substrate. Adhesion proteins are largely absent from the rear of migratory neurons and colocalize significantly with clathrin in the dilation, suggesting that adhesive contacts might be weakened in this domain prior to soma translocation. Pharmacological inhibition of dynamin in neurons migrating in a three-dimensional (3D) ECM substrate produces significant disruptions in migration and alters the subcellular distribution of adhesion molecules. Finally, expressing dominant negative clathrin or dynamin in cortical neurons disturbs migration *in vivo*. These results suggest that endocytosis is critical for normal neuronal migration, and may regulate adhesion to facilitate somal translocation.

## Results

### Components of clathrin-mediated endocytosis are enriched in the dilation of migrating neurons

**Figure 1:**
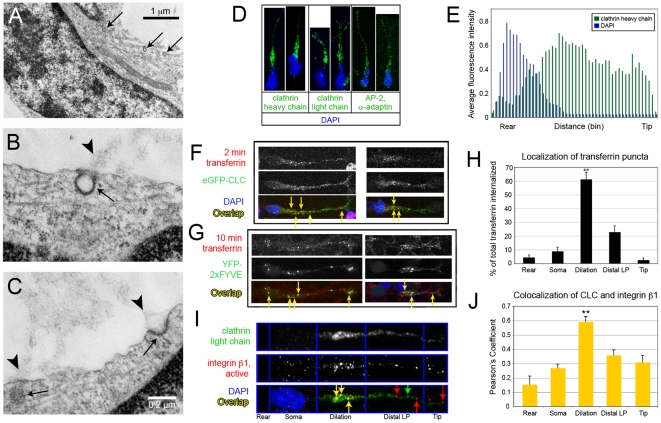
Components of CME present in the dilation of migrating neurons colocalize with adhesion proteins. (A–C) TEM of neurons fixed in the process of migrating reveals CCPs and CCVs (arrows). (A) CCVs near and ahead of the nucleus. Direction of migration is toward the lower righthand corner. (B, C) CCPs at points of ECM attachment (arrowheads). (D) Immunostaining for components of CME (green): CHC (left), CLC (middle), and AP-2 (right). (E) Individual fluorescence intensity line scans were binned to create 50 averaged regions along the length of each measured cell to compare cells of different lengths. Averaged line scan data for CHC (n = 9), normalized for cytoplasm content, shows a peak of intensity in the dilation, just ahead of the nucleus (DAPI, blue). (F) After 2 min exposure, Alexa 594-transferrin partially colocalizes with eGFP-tagged CLC. (G) After 10 min, transferrin partially colocalizes with YFP-2xFYVE+ early endosomes. (H) After 2 min exposure, transferrin is primarily localized to the dilation region. (I) Immunostaining for CLC (green arrows) and an active conformation of integrin β1 (red arrows) show overlap (yellow arrows) in the dilation. (J) Average Pearson's coefficients for each subcellular region (p<0.01, n = 10, mean±SEM, Student's t-test).

In previous studies, we performed correlative electron microscopy on neurons migrating through a three-dimensional (3D) environment [5]. Neurons migrating from anterior subventricular zone (SVZa) explants cultured in Matrigel were followed by time-lapse microscopy until they initiated nucleokinesis. This point in the migration cycle is marked by formation of a transient cytoplasmic dilation in the leading process, just proximal to the cell body. Following dilation formation, explants were rapidly fixed and processed for transmission electron microscopy (TEM). In TEM images of neurons undergoing nucleokinesis, we observed CCVs and clathrin-coated pits (CCPs) in the dilation and perinuclear region (Figure 1A). Clathrin-coated structures (CCSs) were never observed at the tip of the leading process nor at the cell rear. However, CCSs were rare (6 in sections of 5 migrating neurons). Thus, we further examined the localization of clathrin machinery using immunocytochemistry.

Immunostaining for components of CME, including clathrin heavy chain (CHC), clathrin light chain (CLC), and the adaptors AP-2, Dab2, and Numb demonstrated that these components were distributed throughout the leading process (Figures 1D; S1F,I,L). We used the presence of a dilation or an elongated nucleus to identify actively migrating neurons [5], then performed line scan analysis of fluorescence intensity along the cell length and normalized values for cytoplasmic content using genetically-expressed fluorescent proteins. These studies revealed an enrichment of CME components in the dilation (Figure 1E). Because the centrosome and Golgi also occupy the dilation [11], [43], we asked whether clathrin enrichment in this region was solely due to its association with the trans-Golgi network (TGN), which is not involved in surface membrane internalization. TGN-associated clathrin was localized by proximity to staining for GM130, a marker of the Golgi apparatus (Figure S1A). Even after Golgi-proximal clathrin was subtracted from clathrin immunoreactivity, clathrin was enriched in the dilation. Also, CCSs visible by EM were at or near the membrane surface, some in stereotypical shapes indicating an active pinching off process (Figure 1B,C), suggesting that clathrin is involved in surface membrane internalization. Indeed, the cargo-specific adaptor proteins AP-2, Dab2 and Numb were also present in the dilation (Figures 1D; S1F,I,L). These adaptors interact with specific surface molecules to regulate the recruitment of clathrin for cargo-specific internalization [44].

To ascertain whether the dilation is a site of active endocytosis, we exposed cells to Alexa 594-conjugated transferrin, since transferrin-bound transferrin receptors are internalized through CME. This method has been used to demonstrate endocytic hot-spots in dendrites [45]. After 2 min exposure, transferrin puncta were primarily present in the dilation, which contained an average 61.4±5.0% (p<0.001, n = 21 cells) of a cell's total transferrin puncta (Figure 1H), and was colocalized with eGFP-tagged clathrin light chain [46] (Figure 1F). After 10 min (Figure 1G), transferrin colocalized with early endosomes labeled by YFP-2xFYVE [47], demonstrating that internalized transferrin associates with structures later in the endocytic recycling pathway.

Not all endocytic-associated proteins were enriched in the dilation. Caveolin-1, which is involved in caveolae-mediated but not clathrin-mediated endocytosis, was enriched in the tip of the leading process but not the dilation (Figure S1B). These data are consistent with EM of migrating cerebellar granule neurons showing CCVs in the perinuclear region, whereas uncoated vesicles were in the leading process [37]. This could indicate specialized domains for different forms of endocytosis in migrating neurons.

### Clathrin coated vesicles are positioned to regulate adhesion

EM images revealed CCSs at points of ECM contact (Figure 1B,C), suggesting that CME might regulate adhesion between cell membranes and substrates. Indeed, clathrin and dynamin are required for focal adhesion disassembly in model cells migrating in 2D [27], [28], [29]. To test this idea in migrating neurons, we first identified the adhesion receptors used by SVZa neurons to migrate in our ECM substrate. Integrin β1 dimerizes with different integrin α subunits to mediate binding to fibronectin, laminin, or collagen, and is a relevant adhesion receptor in SVZa neurons migrating *in vivo* [48]. Because our substrate is composed primarily of laminin and collagen types I and IV, integrin β1 receptors were likely candidates to mediate adhesion by SVZa neurons *in vitro*. Indeed, culturing explants in the presence of an integrin β1 function-blocking antibody prevented neurons from migrating out of explants (Figure S1C–E).

To examine the subcellular location of CME and adhesion molecules in migrating neurons, we used antibodies to CLC and an active conformation of integrin β1. To quantify colocalization, individual neurons with dilations (thus likely migratory) were divided into 5 regions: leading process tip, distal leading process, dilation, soma, and rear. Each region was examined for colocalization of adhesion and endosome proteins using the JACoP plugin in ImageJ. Consistent with EM images, the highest incidence of colocalization between CLC and active integrin β1 was in the dilation. Pearson's coefficient, a measure of colocalization, was significantly higher in the dilation compared to other subcellular domains (Figure 1J, n = 10, p<0.01, Student's t-test). We also saw greater colocalization between the clathrin adaptor AP-2 and integrin β1 in the dilation than in other regions (Figure S1G, n = 12, p<0.02). Numb, a clathrin adaptor critical for integrin trafficking in other cell types [49], was also more highly colocalized with active integrin β1 in the dilation compared to the cell soma or rear (Figure S1M, n = 6, p<0.02). These observations are consistent with the possibility that endocytosis weakens adhesive contacts in the dilation prior to nucleokinesis. Interestingly, colocalization between Dab2 and integrin β1 in the tip and dilation did not differ significantly, but both sites showed greater colocalization than in other subcellular regions (Figure S1J, n = 9, p<0.04). These data may reflect distinct roles for adaptor proteins in different subcellular domains. Indeed, in migrating HeLa cells, Dab2 maintains an intracellular pool of integrin β1 that can be recycled to create new adhesive contacts at the leading edge [50].

### Inhibiting dynamin function disrupts migration *in vitro*

**Figure 2:**
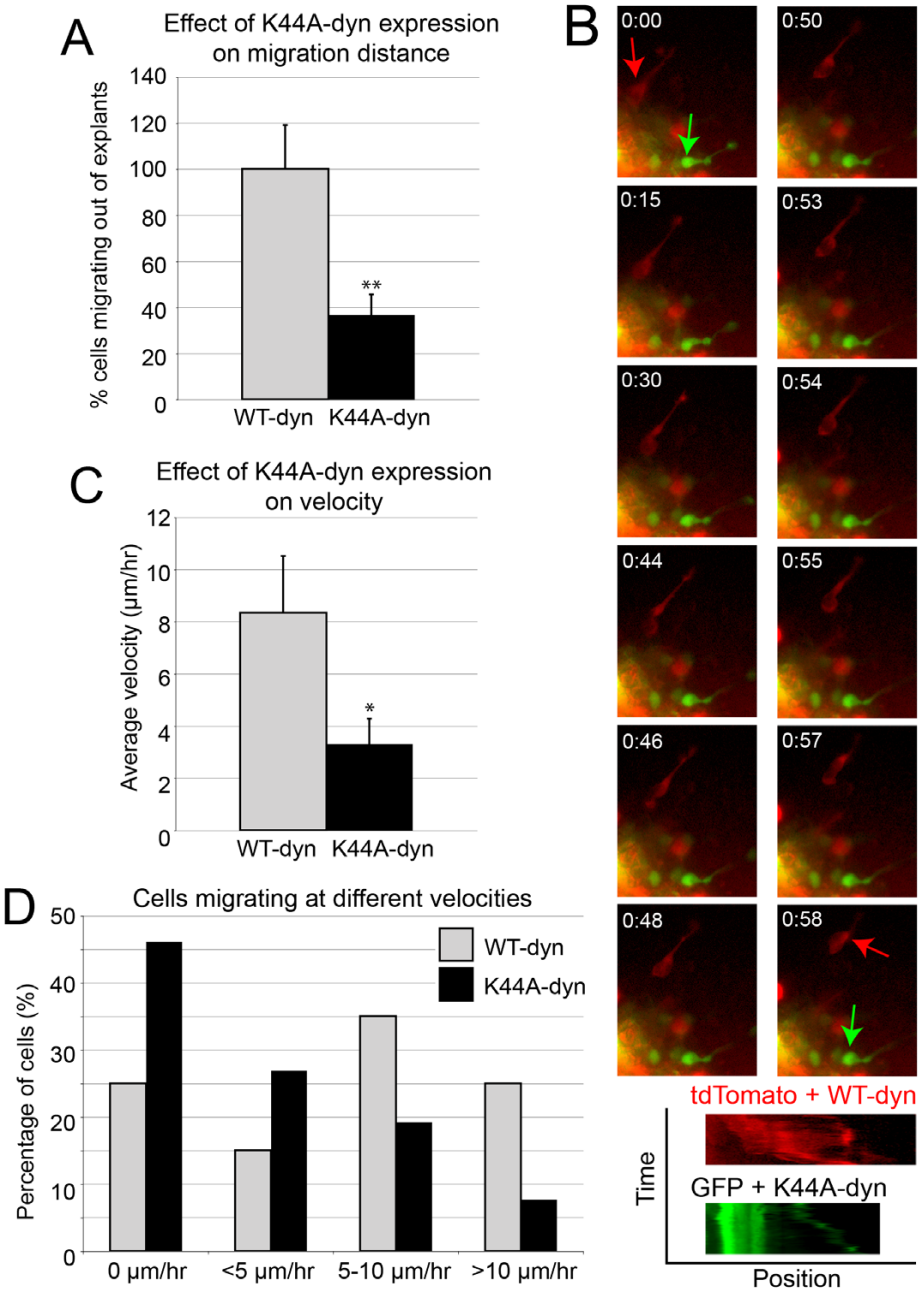
Dominant negative dynamin impairs SVZa neuron migration. (A) Fewer cells migrate out of explants expressing K44A-dyn compared to explants expressing WT-dyn. (B) Example time-lapse series of cells in the same culture expressing either WT-dyn with tdTomato (red arrow) or K44A-dyn with GFP (green arrow). Kymographs showing position (x-axis) over time (y-axis) highlight movement of the cell body. Leading process movement can be seen in the K44A-dyn/GFP cell. See Movie S1. (C) K44A-dyn expressing cells that migrate out of explants have a significantly lower migration rate than WT-dyn expressing cells. (D) A greater percentage of K44A-dyn cells do not migrate, or have slower rates than those expressing WT-dyn. Data presented as mean±SEM, *p<0.05, **p<0.01, Student's t-test.

The association between CCSs and adhesions in the dilation and relative dearth of adhesion puncta at the rear of migrating neurons (discussed later in the manuscript) suggest that endocytosis may be involved in removing or weakening adhesions prior to somal translocation. If true, blocking endocytosis might: 1) prevent neurons from migrating or cause them to migrate more slowly; and 2) alter the subcellular distribution of adhesion molecules such that adhesions accumulate at the cell rear. To ascertain whether inhibiting endocytosis affects neuronal migration, we expressed dominant negative dynamin (K44A-dyn) [51] in SVZa neurons migrating out of explants. The K44A substitution disrupts the GTPase activity of dynamin and has been used frequently to block endocytosis [52], [53], [54]. Significantly fewer cells migrated out of explants expressing K44A-dyn (36.7±8.8% of WT-dyn, p<0.01, n = 3 experiments, 32–39 explants) (Figure 2A). Cells that did migrate out of explants exhibited altered dynamics (Figure 2B,C; Movie S1). The average velocity of K44A-dyn cells observed was 3.3±1.0 µm/hr (n = 26 cells), which was significantly (p<0.05, Student's t-test) slower than average WT-dyn cell velocity (8.3±2.2 µm/hr, n = 21 cells). More neurons expressing K44A-dyn failed to migrate at all, but those that did move had slower rates than cells expressing WT-dyn (Figure 2D).

To explore the role of dynamin on migration dynamics with finer temporal resolution, we cultured explants in the presence of myristyl trimethyl ammonium bromide (MiTMAB), a small molecule inhibitor of dynamin [55]. Transferrin internalization in neurons exposed to MiTMAB was assessed by adding Alexa 594-conjugated transferrin for 15 min to explants cultured in 1% DMSO (vehicle control) or 10, 30, 50, or 100 µM MiTMAB; explants were exposed to MiTMAB for a total of 30 min. Transferrin internalization at 10 and 30 µM MiTMAB did not differ significantly from vehicle-treated explants, but 50 µM MiTMAB reduced transferrin internalization to 72.3±9.9% (p<0.01, Student's t-test) (Figure S2A) without affecting bulk fluid-phase endocytosis (as measured by Texas Red-dextran internalization, Figure S2B). In addition, MiTMAB treatment at 30 µM significantly increased the surface levels of integrin β1 (Figure S2C).

**Figure 3:**
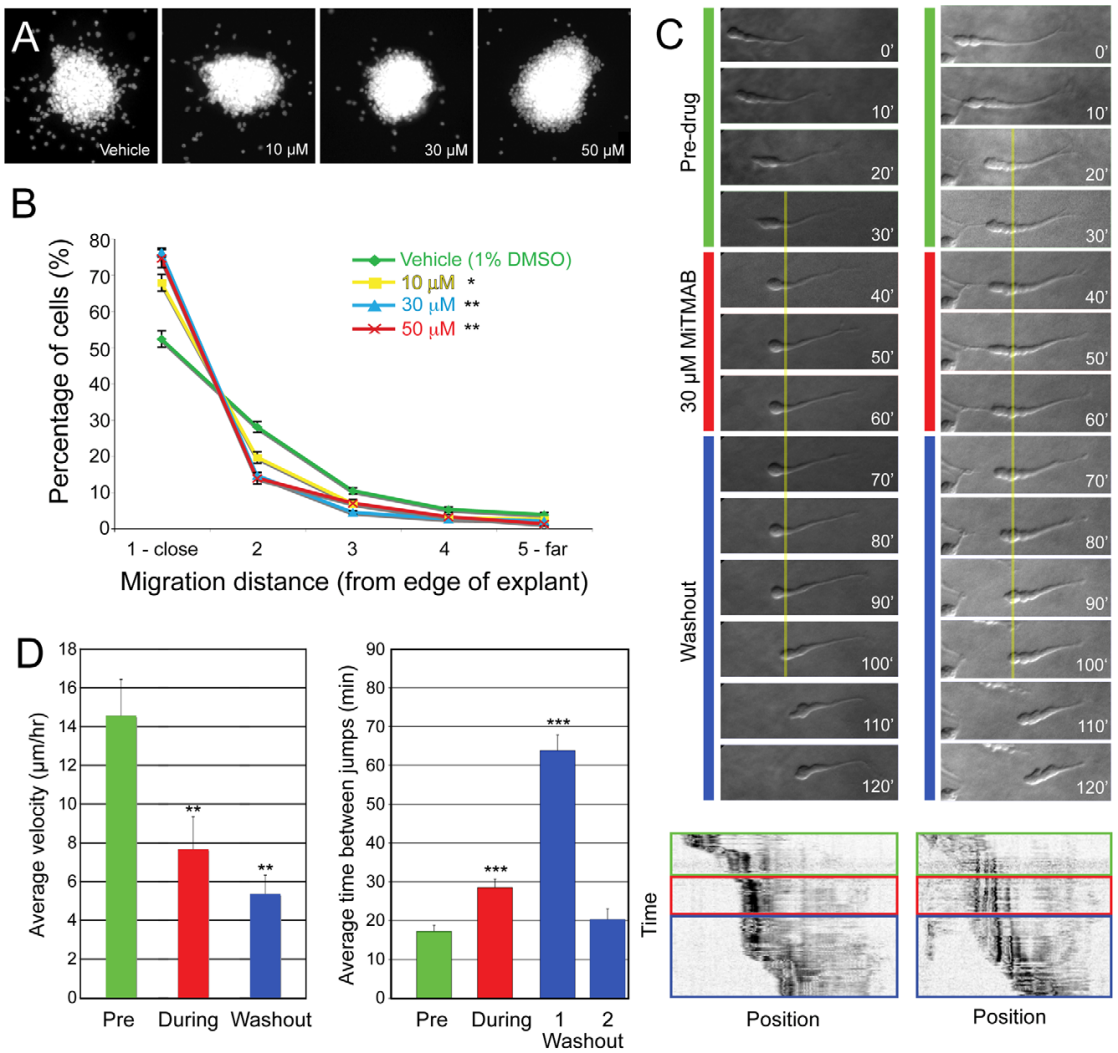
Inhibiting dynamin impairs SVZa neuron migration. (A) Images of explants treated with varying concentrations of MiTMAB. Fewer DAPI-stained nuclei are present outside explants at increasingly higher concentrations of MiTMAB. (B) Effect of MiTMAB treatment on the percentage of cell nuclei present in evenly-spaced concentric rings surrounding the explant at increasing distances. (C) Time-lapse series of two neurons treated with MiTMAB. The yellow line marks the position of the dilation at the time of MiTMAB addition, which becomes that of the cell rear after washout. (Bottom) Kymographs plotting time vs. position highlight the positions of the cell rears. See Movies S2, S3. (D) Average velocity (µm/hr) of neurons migrating before the addition of MiTMAB is significantly faster than that in the presence of the drug or after washout (n = 19). The amount of time a cell spends in the same position between translocations is significantly longer before the first translocation after washout (Washout-1, n = 16) compared to either before (Pre, n = 15) or in the presence of MiTMAB (During, n = 13). Cells that made additional translocations during washout (Washout-2, n = 6) showed inter-translocation interval values similar to those before MiTMAB addition. Data presented as mean ± SEM, *p<0.05, **p<0.01, ***p<0.001, Student's t-test.

MiTMAB was then applied to SVZa neurons migrating in a 3D matrix for at least 4 hours, which decreased the distance over which neurons migrated from the edge of explants (Figure 3A,B). The sensitivity of migration to MiTMAB treatment exceeded that shown by transferrin internalization, since migration *in vitro* was significantly altered by treatment with only 10 µM MiTMAB (Figure 3B). Effects on surface integrin levels also began to appear at 10 µM, with a significant increase seen at 30 µM (Figure S2C). This suggests that transferrin internalization may not appropriately represent the kinetics of endocytosis for adhesions. Indeed, integrin internalization requires adaptor proteins distinct from those used for transferrin receptor endocytosis [49], [50], and different receptors internalized through CME take different pathways [44]. It is unlikely that the effect of MiTMAB on SVZa cell migration is due to a general disruption in bulk endocytosis, since MiTMAB did not affect dextran internalization at lower concentrations (Figure S2B).

### Acutely inhibiting dynamin function disturbs cell soma translocation

Using pharmacological inhibitors on neurons migrating *in vitro* affords the opportunity to disrupt endocytosis acutely and observe consequent migratory behavior. SVZa neurons migrating from explants were imaged using differential interference contrast (DIC) time-lapse microscopy, and the regular imaging medium was replaced with MiTMAB-containing medium. Cells were visualized for 30 min during MiTMAB treatment, then the medium was replaced with normal imaging medium (washout). In the presence of MiTMAB, neurons typically paused and failed to move forward, or made a single somal translocation near the beginning of treatment (Figure 3C; Movies S2,S3). Average velocity was greatly reduced during MiTMAB treatment (7.6±1.7 µm/hr, n = 19, p<0.01, Student's t-test) compared to the rate prior to drug addition (14.5±1.9 µm/hr) (Figure 3D). Time between somal translocations was also significantly increased during treatment (Figure 3D). Leading process dynamics appeared generally unaffected, as growth cones continued to make exploratory movements in the presence of MiTMAB.

The changes in migration rate and time between translocations were even more exaggerated immediately after washout of MiTMAB (Figure 3D, Washout-1). The average rate of migration during this period was 5.4±1.0 µm/hr, significantly slower than before MiTMAB addition (p<0.001). On average, neurons remained stationary for 63.8±4.0 min before moving forward immediately after washout, more than 3 times as long as before and twice as long as during MiTMAB treatment (p<0.001). When cells did eventually move their somata forward, the rear membrane often required a longer period of time to resolve; kymographs revealed that the movement of cell rears formed a sloped line rather than a discrete jump (Figure 3C). Sustained effects after washout may be due to disrupting adhesion removal in the dilation present during treatment. This is predicted to interfere with cell soma translocation in the next cycle of migration, which would occur after washout. Consistent with this interpretation, cells imaged for longer periods after MiTMAB washout appeared to recover, as the time between translocations returned to baseline levels (20.2±2.8 min, n = 6) for cells that were imaged long enough to observe additional translocations (Figure 3D, Washout-2).

### Inhibiting clathrin function disrupts migration *in vitro*

**Figure 4:**
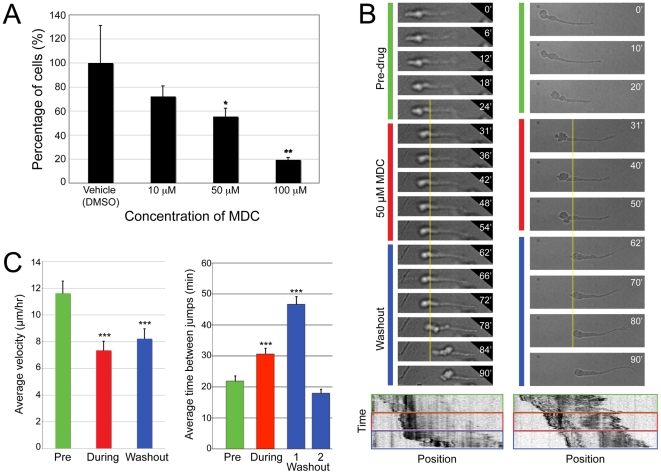
Inhibiting clathrin impairs SVZa neuron migration. (A) Fewer DAPI-stained nuclei are present outside explants at increasingly higher concentrations of MDC. (B) Time-lapse series of two neurons treated with MDC. The yellow line marks the position of the dilation at the time of MDC addition, which becomes that of the cell rear after washout. (Bottom) Kymographs plotting time vs. position highlight the positions of the cell rears. See Movies S4, S5. (C) Average velocity (µm/hr) of neurons migrating before the addition of MDC is significantly faster than that in the presence of the drug or after washout (n = 63). The amount of time a cell spends in the same position between translocations is significantly longer before the first translocation after washout (Washout-1, n = 66) compared to either before (Pre, n = 48) or in the presence of MDC (During, n = 40). Cells that made additional translocations during washout (Washout-2, n = 46) showed inter-translocation interval values similar to those before MDC addition. Data presented as mean ± SEM, *p<0.05, **p<0.01, ***p<0.001, Student's t-test.

To ask whether the effects of inhibiting dynamin were a result of disrupting CME, we applied the pharmacological clathrin inhibitor monodansyl cadavarine (MDC) [56] to migrating neurons. MDC inhibits clathrin-mediated receptor internalization by stabilizing clathrin cage assembly, preventing clathrin polymerization [57], [58]. MDC prevented SVZa neurons from migrating out of explants in a dose-dependent manner (Figure 4A) that corresponds to its ability to inhibit transferrin internalization (Figure S3A). Although higher concentrations of MDC prevent integrin β1 and L1 internalization in HEK cells [33], MDC did not significantly affect surface integrin β1 levels in SVZa neurons migrating in Matrigel (Figure S3C). The effect of MDC on migration dynamics differed slightly from those of MiTMAB; although migration was slowed in the presence of MDC, the velocity after washout did not show a further decrement (Figure 4C). The time between translocations was increased for the translocation event immediately following washout (Figure 4D, Washout-1), but returned to baseline for subsequent translocations (Figure 4D, Washout-2), similar to MiTMAB treatment. The differences between the effects of MiTMAB and MDC may reflect distinct functions for clathrin-dependent vs. -independent mechanisms of migration. Alternatively, neurons may rapidly compensate for a block in clathrin-dependent forms of endocytosis by utilizing clathrin-independent but dynamin-dependent endocytosis, such as that mediated by caveolae.

### Inhibiting dynamin function alters cell morphology and the distribution of adhesions

**Figure 5:**
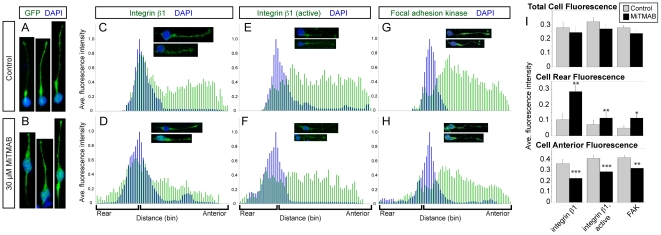
Inhibiting dynamin alters the morphology of and distribution of adhesion proteins in migrating neurons. (A,B) Cytoplasmic GFP and DAPI reveal the morphology of neurons treated with (A) 1% DMSO vehicle or (B) MiTMAB. (C–H) Average line scans of fluorescence intensity normalized by cytoplasmic GFP were aligned by nuclear position, as determined by maximum scaled DAPI value. Integrin β1 (C, control, n = 9; D, MiTMAB, n = 8), active integrin β1 (E, control, n = 7; F, MiTMAB, n = 6), and FAK (G, control, n = 9; H, MiTMAB, n = 10). (I) Average fluorescence intensity of adhesion markers for: (top) all bins across cell position; (middle) all bins posterior to maximum DAPI value; (bottom) all bins anterior to maximum DAPI value. Data presented as mean ± SEM, *p<0.05, **p< 0.01, ***p<0.001, Student's t-test.

The time-lapse analyses above are consistent with the hypothesis that dynamin-mediated endocytosis regulates cell soma translocation in migrating neurons. To ascertain whether MiTMAB also disrupts the ability of cells to release from substrates at the cell rear, we examined the morphologies of MiTMAB-treated SVZa neurons. In normal SVZa neurons, few migrating cells retain a trailing process (Figure 5A). However, neurons exposed to MiTMAB for at least 2 hours were more likely to exhibit a trailing process or tail (Figure 5B). Similar bulbs of membrane and cytosol at the cell rear are visible transiently in normal migrating SVZa cells but these usually resolve quickly, presumably due to myosin II-mediated contraction during somal translocation [5], [12].

The presence of membrane tails in MiTMAB-treated neurons suggests that the rear membrane was impeded in detaching from the substrate, and that there might be a concomitant accumulation of adhesion molecules at the cell rear. While adhesion proteins were typically present in the leading process but not the rear of control SVZa neurons (Figure 5C,E,G; S4A, B, C), these proteins were seen in the rear regions of neurons exposed to MiTMAB for at least 2 hrs (Figure 5D,F,H; S4D, E, F). Quantification revealed a significant increase in the average normalized fluorescence intensity for all bins posterior to the position of maximal fluorescence intensity in the nucleus in MiTMAB-treated cells (Figure 5I), and the rearward redistribution of adhesion proteins occurred whether or not the neuron displayed a trailing process. This increase in adhesion protein presence at the cell rear was not due to an overall increase in fluorescence intensity, as the total average normalized fluorescence intensity was not significantly different between control and MiTMAB-treated cells (Figure 5I). However, the average normalized fluorescence intensity for bins anterior to the position of maximal fluorescence intensity in the nucleus in MiTMAB-treated cells was significantly lower than that of control cells, suggesting a rearward redistribution rather than general alteration in adhesion protein levels. These data are consistent with the hypothesis that endocytosis in the dilation removes or weakens adhesions prior to somal translocation and may contribute to the slowed migration of these neurons.

### Inhibiting clathrin function delays cortical migration *in vivo*

Although different subtypes of neurons use distinct substrates and modes of migration, they follow the same general sequence of steps in the migration cycle, including the formation of a cytoplasmic dilation [7], [59]. Because CME may be important for regulating adhesion at this critical step, we examined whether endocytosis plays a role in the migration of neurons *in vivo*, using neurons of the developing cerebral cortex.

**Figure 6:**
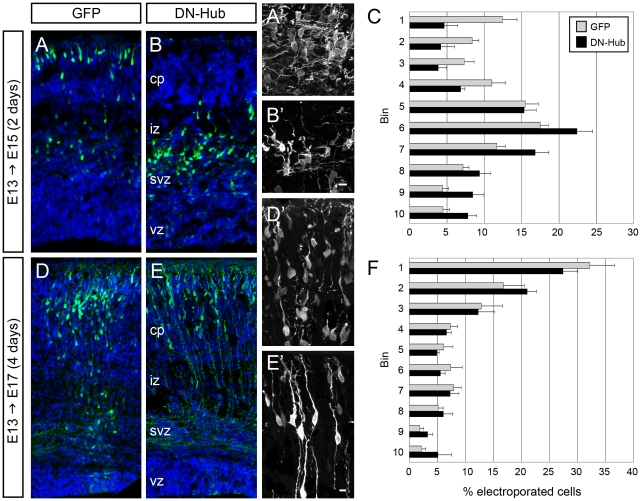
Dominant negative clathrin expression leads to delayed cortical migration. (A–C) After 2 days migrating in the developing cortex, neurons expressing DN-Hub (B) did not populate the CP as did control cells expressing GFP only (A). DN-Hub-expressing cells in the IZ/SVZ (B′) show similar morphology to GFP-only-expressing cells (A′). (C) The distribution of cells expressing DN-Hub vs GFP differs significantly at E15 (p<0.01, Chi-square test). DN-Hub-expressing neurons in their final position in the CP (E, E′) resemble GFP-only expressing neurons (D, D′). (F) The distributions of cells expressing DN-Hub vs GFP do not differ significantly at E17 (Chi-square test). Mean ± SEM. Scale bars, 10 µm.

Either GFP alone or GFP along with a dominant negative clathrin construct (DN-Hub) was introduced into developing mouse cortices at E13.5 using in utero electroporation. DN-Hub encodes the Hub region of CHC, which binds to CLC to prevent the regulated formation of the clathrin coat [60]. Electroporated embryos survived for either 2 days (E15.5) or 4 days (E17.5) and were then processed for immunohistochemistry (Figure 6). To determine the distribution of GFP+ cells, images of cortex were divided into 10 evenly-spaced bins from the ventricular to the pial surface and the number of GFP+ neurons was counted in each bin. After 2 days survival, 28.2±2.9% (n = 7 brains, 4574 cells) of neurons expressing only GFP had reached the cortical plate (CP, bins 1–3) (Figure 6A,C). The distribution of neurons expressing DN-Hub differed significantly (n = 8 brains, 7615 cells, p<0.02, Chi-square test), with only 12.7±4.6% of neurons present in the CP (Figure 6B,C). Most were in the intermediate and subventricular zones (IZ/SVZ, 70.9±4.2%, bins 4–8). After 4 days survival, however, the distribution of DN-Hub expressing cells did not differ significantly from that in controls (p>0.5, Chi-square test, n = 4 GFP brains, 1945 cells and 7 DN-Hub brains, 3297 cells), suggesting that cell migration had recovered (Figure 6D–F). The morphology of DN-Hub expressing neurons appeared normal for their positions; cells in the IZ had a multipolar morphology similar to GFP-only cells in transit through the IZ (Figure 6A′,B′), while cells that settled in the CP had similar morphology to GFP-only cells in the CP (Figure 6D′,E′).

The ability of neurons transfected with DN-Hub to finally migrate into the CP was not due to a loss of DN-Hub expression. Staining for a T7 tag in the DN-Hub construct revealed that many GFP+ cells showed similarly strong T7 staining at 2 and 4 days after electroporation, including cells that migrated into the CP (Figure S5A–F). The delay in migration caused by DN-Hub was not due to a disruption of radial glia morphology or the structural integrity of the apical surface, since both appeared normal in electroporated brains (Figure S6A–D and not shown).

### Inhibiting dynamin function stalls cortical migration *in vivo*

To independently probe the role of endocytosis in cortical migration, we introduced a dominant negative dynamin I (K44A-dyn) construct [51] into E13.5 mouse cortices using in utero electroporation. Either K44A-dyn or a control wild-type dynamin I construct (WT-dyn) was co-electroporated with GFP. Embryos survived for 2 or 4 days and the position of GFP+ neurons in the cortex was determined as described above.

**Figure 7:**
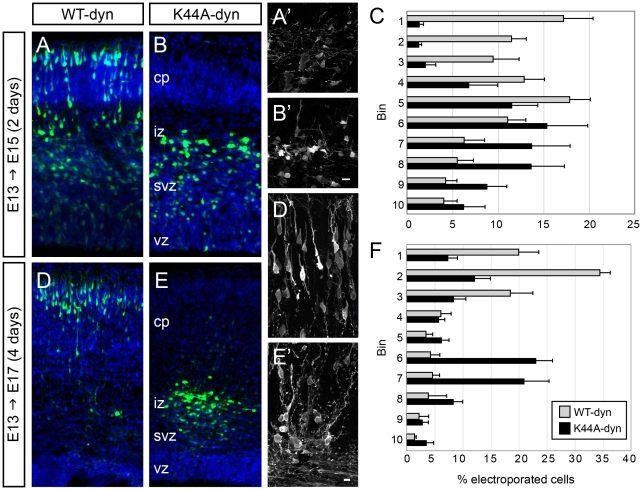
Dominant negative dynamin expression leads to stalled cortical migration. (A–C) Neurons expressing K44A-dyn (B,E) did not reach the CP after 2 days migrating in the developing cortex, unlike cells expressing WT-dyn (A,D). Higher power view of WT-dyn (A′) and K44A-dyn (B′) cells in the IZ at E15, and of WT-dyn cells in the CP (D′) and K44A-dyn cells in the IZ (E′) at E17. (C, F) The distributions of cells expressing K44A-dyn at E15 and E17 differ significantly from those of WT-dyn-expressing cells (p<0.01, Chi-square test). Mean ± SEM. Scale bars, 10 µm.

After 2 days, neurons expressing WT-dyn had reached the top of the CP (Figure 7A,C, n = 5 brains, 3740 cells), similar to neurons expressing GFP only in the previous experiment (Figure 6A). In contrast, the majority of cells expressing K44A-dyn were in the IZ/SVZ (Figure 7B,C; n = 5 brains, 3386 cells). Unlike cells expressing DN-Hub, after 4 days of survival, cells expressing K44A-dyn did not fully recover to match the positions of control WT-dyn cells; the distribution of K44A-dyn cells at E17 differed significantly from that of WT-dyn cells (p<0.001, Chi-square test) (Figure 7E,F). At this stage, 53.0±5.8% (n = 7 brains, 1869 cells) of K44A-dyn-expressing neurons were present in the IZ/SVZ, compared to 18.7±7.5% (n = 3 brains, 1576 cells) of WT-dyn-expressing neurons (Figure 7D,E). GFP+ cells transfected with K44A-dyn that reached the top of the CP still expressed the DN construct, assayed by continued expression of the HA-tag (Figure S5G–R), but the intensity of staining in these neurons was lower than in cells in the IZ/SVZ (Figure S5Q). This difference suggests that neurons that were able to migrate to the CP expressed lower levels of K44A-dyn. Alternatively, cells that reached the CP may have utilized a dynamin-independent form of endocytosis. Both radial glia morphology and the integrity of the ventricular surface appeared normal in electroporated brains (Figure S6E–H and not shown), suggesting that the disruption in migration reflects a requirement for dynamin in migrating neurons.

The morphology of K44A-dyn-expressing neurons in the IZ/SVZ at E15 (Figure 7B′) appeared similar to that of cells expressing WT-dyn (Figure 7A′). At E17, although most K44A-dyn-expressing neurons were in the IZ, they no longer displayed the morphology of multipolar migrating neurons but instead extended neurites (Figure 7E′) and expressed Tuj1, a neuronal-specific β-tubulin isoform (not shown), suggesting that they differentiated within the IZ. In parallel experiments, cortical neurons electroporated with K44A-dyn at E13.5 and examined at P1 (Figure S7) expressed K44A-dyn, as assessed by HA staining, and were still present in the white matter (Figure S7B,E). The effect of dynamin disruption appeared to be selective for migration, since the transfected cells survived, differentiated (Figure S7B, inset), and extended axons across the corpus callosum (Figure S7D), similar to control cells (Figure S7C).

## Discussion

Attachment and detachment are critical steps in cell migration. Here we find pronounced differences in the subcellular localization of adhesion molecules in migrating neurons: adhesions are present in the leading process but sparse or absent at the cell rear. EMs revealed CCVs at points of adhesive contact with a matrix substrate, and immunostaining confirmed that CME components colocalize with adhesion receptors in the dilation of migratory neurons. Dominant negative and pharmacological approaches that interfere with endocytosis disrupted neuronal migration both *in vitro* and *in vivo*. Inhibiting dynamin function *in vitro* caused a redistribution of adhesion molecules to the cell rear, consistent with the notion that CME in the dilation weakens adhesions to facilitate forward translocation during neuronal migration.

### Spatial regulation of adhesion and de-adhesion

It has long been appreciated that there are distinct zones of adhesivity in polarized migrating cells. Nascent adhesions at the leading edge provide strong traction forces [61]. Small yet strong focal complexes can then either undergo turnover or mature into larger focal adhesion structures [23]. Focal adhesions at the cell rear transmit less force from substrate to cytoskeleton [62], [63]. Collectively these processes establish the differential adhesive strengths required for forward movement. Although adhesion molecules in neurons were distributed in small puncta rather than identifiable focal adhesion complexes, they also displayed distinct zones that may reflect differential adhesivity. We speculate that adhesions at the leading edge are used for traction, while those in the dilation create an intermediate anchor point toward which the nucleus can move. The dilation of migrating neurons is reminiscent of the “culling zone” between the nucleus and tail of migrating fibroblasts, where focal adhesions are disassembled [64] and which becomes the location of the cell rear in the next cycle of migration.

Regulation of adhesion assembly and disassembly mediates the creation of asymmetric zones of adhesion in migrating cells. Adhesion turnover can occur through biochemical mechanisms such as phosphorylation/de-phosphorylation and proteolysis [15], [24], [65], biomechanical breaks generated by acto-myosin contractile forces [66], [67], or by endocytosis [27], [28], [29]. Endocytosis is a particularly attractive candidate to regulate adhesions in light of the long-standing idea that integrin receptor recycling to a cell's leading edge provides both membrane and adhesion receptors to facilitate continued forward migration [68], [69], [70], [71]. Although there is no direct evidence that integrins internalized at the rear are re-inserted at the front of migrating cells, integrin receptors do travel in endosomes in many cell types and their recycling is important for movement [72], [73], [74], [75]. In fibroblasts, loss of the EHD1 protein, which regulates integrin β1 endosomal transport, results in slowed focal adhesion disassembly, larger focal adhesions and impaired migration [76]. The clathrin adaptor Dab2 appears to be involved in trafficking integrin receptors to mediate its role in migration [50]. Here we find that Dab2 and integrin β1 colocalize in the dilation and tip of the leading process (Figure S1I), suggesting that Dab2 may also be involved in this process in neurons. There is also evidence for asymmetric L1 adhesion molecule endocytosis in subdomains of the axonal growth cone [30], [31], [32]. A recent model proposes that polarized membrane trafficking is coordinated through both caveolar- and clathrin-mediated endocytosis in specialized subcellular domains [77].

Our studies provide three lines of morphological evidence that CME is involved in regulating the distribution of adhesion molecules in neurons. First, EM images suggest that CCSs are located preferentially at points of contact between the membrane of migrating neurons *in vitro* and the surrounding ECM. Second, immunostaining reveals that adhesion molecules and CME components colocalize preferentially in the dilation of neurons migrating *in vitro*. Third, adhesion components accumulate at the cell rear when dynamin-dependent endocytosis is disrupted *in vitro*.

These data are consistent with the hypothesis that endocytosis plays a role in regulating adhesion in migrating neurons, but do not rule out other roles for endocytosis or a role for other membrane traffic pathways mediated by clathrin and dynamin. For example, CME drives the polarized trafficking of growth factor BDNF in the directed migration of cerebellar granule cells [78], and axon growth cone dynamics rely on both endo- and exocytosis in regulating turning responses to guidance cues [30], [31]. However, growth cone motility may utilize a clathrin-independent, bulk endocytosis pathway rather than CME [79]. In our studies, MiTMAB blocked somal translocation *in vitro* but not leading process dynamics, suggesting that alternative forms of endocytosis may regulate adhesion at the growth cone vs. cell soma.

### Adhesion *in vivo*

Neurons can migrate along a variety of substrates: radial glia, other neurons, and ECM. The adhesion molecules that mediate migration differ depending on the substrate. Integrins connect the cytoskeleton to ECM and, though critical for cortical neuron migration [80], [81], [82], other molecules (e.g. astrotactin, connexins, or PSA-NCAM) play key roles in radial and chain migration that depend on cell-cell interactions for movement [38], [83], [84]. Despite the diversity of molecular players, all cells must regulate adhesion, and EM observations reveal CCVs near adhesive contacts in many types of migrating neurons, including cerebellar granule neurons migrating along glia [36], [37] and SVZa neurons undergoing chain migration [38]. Interestingly, CCVs are present in the perinuclear region of migrating granule neurons whereas uncoated vesicles are enriched at the leading process tip [37], supporting the idea that distinct forms of endocytosis may regulate movement at the leading edge vs. cell rear.

### The role of clathrin-mediated endocytosis in neuronal migration

Altering the balance of adhesion strength in either direction (causing weaker or stronger adhesions) disrupts the migration of model cells *in vitro* [22], [66]. Here we show that disrupting either clathrin or dynamin *in vivo* slows or stops the migration of young neurons and causes defects in cell soma translocation. A number of recent studies support our findings on dynamin's importance in neuronal migration. A study of somal translocation in serotonergic neurons found that disrupting dynamin impacted cell soma movement and decreased migration velocity [85]. In glial-guided migrating cerebellar granule neurons, dynamin-dependent surface adhesion may be regulated by astrotactin family members [86]. Both cortical neuron radial migration and N-cadherin distribution are affected by disruptions in Rab GTPase-dependent endocytic trafficking [35]. These observations are consistent with the possibility that increased adhesion at the cell rear might lead to an inability of affected cells to detach from their migratory substrates. Our experiments revealed that neurons treated with MiTMAB *in vitro* accumulated adhesion proteins at the cell rear and were more likely to exhibit tails.

One might argue that the effects of inhibiting dynamin, whether through K44A-dyn or MiTMAB, were not due to disrupting endocytosis but instead reflect the role of dynamin in cytoskeletal remodeling [87]. However, we saw no overt alterations in the organization of actin or microtubules in MiTMAB-treated neurons (data not shown), apart from the failure of migrating neurons to resolve their trailing processes at the cell rear. In addition, disrupting clathrin function through RNAi-mediated CHC knockdown (data not shown), DN-Hub, or MDC also impairs neuron migration, implicating CME in cell movement. Nevertheless, adhesion regulation is tightly linked to cytoskeletal dynamics. Actin is involved in recruiting adhesion components to integrin receptor complexes, and microtubules target focal adhesions and induce dynamin- and clathrin-dependent disassembly [27], [29]. Thus, if dynamin does directly affect cytoskeletal dynamics in migrating neurons, this role is likely one facet of a coordinated system for regulating adhesion and polarity in directed cell migration.

Our *in vitro* and *in vivo* experiments both showed more severe effects of disrupting dynamin compared to clathrin, and suggest distinct roles for endocytosis in regulating somal translocation. Disrupting clathrin with MDC or dynamin with MiTMAB each led to an immediate decrease in neuronal migration velocity *in vitro*. However, MiTMAB but not MDC treatment led to longer-term alterations in morphology and adhesion distribution. We suspect that the immediate effect of disrupting endocytosis was not due to changes in adhesion, but rather an effect on the mechanics of somal translocation, perhaps force-generating membrane internalization. The delayed effect of altering adhesion in the dilation would appear in later cycles of migration when the dilation has become the new cell rear. Indeed, MiTMAB-treated cells showed a more extreme slowing of migration over time, the formation of cell tails, and a rearward shift in adhesion protein localization, whereas MDC-treated cells did not. It is possible that MDC-treated cells compensated by utilizing a clathrin-independent, dynamin-dependent form of endocytosis, such as that mediated by caveolin. Consistent with this notion, caveolin-1 staining appeared to shift from the leading process tip to a more even distribution throughout migratory cells after MDC treatment (not shown).

*In vivo* cortical migration was disrupted more dramatically in response to expression of dominant negative dynamin compared to dominant negative clathrin. Multiple endocytic pathways exist for internalizing molecules into a cell and there are numerous reports of instances in which secondary endocytic routes dominate when primary routes are blocked [88], [89]. Neurons expressing DN-Hub may have compensated for the disruption of clathrin formation by utilizing alternate forms of endocytosis such as caveolae-based internalization, which is clathrin-independent [90]. Because most other forms of endocytosis do require dynamin function, K44A-dyn expressing neurons might show a more severe phenotype due to an inability to compensate with alternative mechanisms. However, clathrin-independent, dynamin-independent pathways do exist, since HeLa cells can switch to a clathrin- and dynamin-independent form of endocytosis after only 30–45 minutes of overexpressing dominant negative dynamin [91]. However, the molecular mechanism of this novel form of endocytosis is still unknown [89] and whether such a pathway exists in neurons is not clear.

### A model for neuronal migration

**Figure 8:**
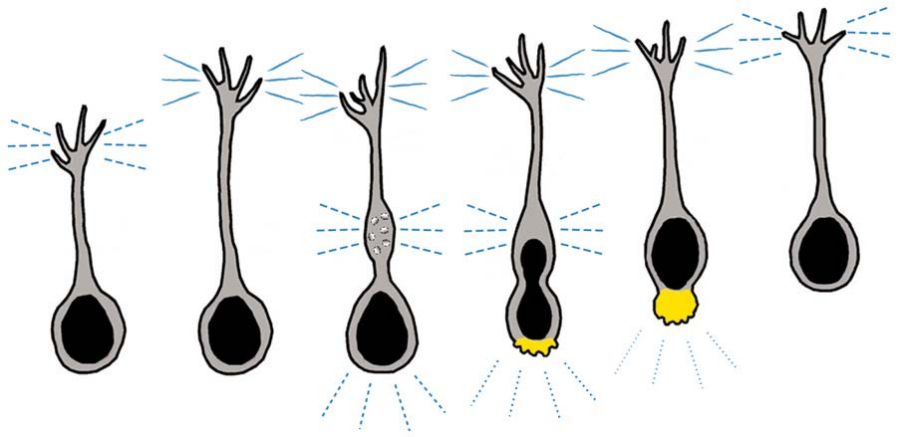
Model of Neuronal Migration. A proposed model of neuronal migration consistent with our findings, though future work should address adhesion dynamics directly. Neurons first extend an exploratory leading process that forms transient and dynamic adhesions (perforated blue lines) in the growth cone. The leading process pauses, strengthening adhesions (solid blue lines) to provide traction. A cytoplasmic dilation forms, where endocytosis (gray circles) weakens adhesion. Myosin II (yellow) mediated contractions at the rear squeeze the nucleus forward and disrupt remaining attachments. Nucleokinesis into the former dilation establishes a new cell rear.

Based on previous research and our current results, we propose the following model of directed neuronal migration (Figure 8). When a neuron receives a guidance cue to migrate in a particular direction, the cell polarizes and forms a single leading process, which explores the substrate. Eventually, anchoring adhesions form in the growth cone to provide traction forces. To ensure that a gradient of adhesive strength is maintained to enable the cell rear to detach from the substrate during somal translocation, endocytosis in the dilation may weaken adhesive contacts and prepare the cell for nuclear translocation into the dilation, which defines the new cell rear. Somal translocation is driven by myosin II contractions behind the nucleus [5], [12], which coordinate with dynein at the centrosome to move the nucleus into the dilation [59], [92]. Contractions at the cell rear likely break any remaining contacts between the substrate and cell membrane during nuclear movement and thus retract the rear membrane. Our data is consistent with, though does not prove, the idea that endocytic events in the dilation enable the nucleus to move into a region in which attachments have already been weakened, thus facilitating the next cycle of somal translocation. To further examine the validity of our model, future experiments should utilize dynamic imaging of adhesions in migrating neurons, disruption of endocytosis through the directed application of inhibitors at the dilation, either by pipet or caged inhibitors, and further study of adhesions *in vivo*.

Although this model is primarily based on observations of SVZa neurons in culture, SVZa cells migrating in chains *in vivo* and cortical neurons migrating on radial glia undergo a similar sequence of morphological changes during movement [8], [9], [93] that appear to utilize common cytoskeletal mechanisms [94]. However, neurons migrating *in vivo* may require a distinct mechanism to regulate adhesion at the rear if they leave a trailing process or grow an axon during migration. For example, in migrating SVZa neurons and tangentially migrating interneurons, which do not extend an obvious trailing process during migration, myosin II at the rear of the cell aids in somal translocation [5], [11]. However, cerebellar granule neurons, which extend an axon as they migrate, show myosin II activation ahead of the nucleus in the dilation region or in the growth cone [7], [95]. Further explorations of the commonalities and differences between distinct modes of migration will enhance our understanding of how neurons reach their final positions during development.

### Broader implications for three-dimensional adhesion systems

Historically, studies of the cell biology of migration have focused on cells migrating on 2D substrates. However, when those same types of cells are examined migrating in 3D substrates that more closely mimic the *in vivo* environment, the distribution, composition, size, and dynamics of those adhesions are different [96], [97]. The morphology of cells migrating in 3D can differ from the same cell type migrating in 2D [98], [99], and some cell types resemble migrating neurons in their morphology when they migrate in 3D [100]. Indeed, the diffuse adhesions observed in migrating neurons resemble the smaller focal contacts called “3D-matrix adhesions” seen in fibroblasts cultured in 3D environments [96]. This raises the possibility that what we have learned here from migrating neurons may also be applied to more general cell biology. Future work to test the role of endocytosis in adhesion regulation in other types of migrating cells will facilitate an understanding of how the cytoskeleton is coordinated to enable cell migration.

## Materials and Methods

### Ethics Statement

All animals were treated under protocol #11499 approved by Stanford University Institutional Animal Use and Care Committee.

### Materials: chemicals, plasmids, antibodies

Myristyl trimethyl ammonium bromide (MiTMAB) and monodansyl cadavarine (MDC) were from Sigma Aldrich. All chemicals for EM were from Electron Microscopy Services (Fort Washington, PA).

Plasmids containing HA-tagged wild-type (WT-dyn) and dominant-negative (K44A-dyn) dynamin I were gifts from Sandra Schmid. The plasmid containing T7-tagged dominant-negative Hub (DN-Hub) was generated as described [60]. TdTomato and mCherry were gifts from Roger Tsien [101]. All constructs were subcloned into a pCDNA3.1 vector with the CA (CMV promoter with chicken β-actin enhancer) promoter and verified by DNA sequencing. For expression of GFP only, GFP under the CA promoter in a pBlueScript vector was used. YFP-2xFYVE construct was a gift from Harald Stenmark [47]. eGFP-FKBP-clathrin light chain construct was a gift from Timothy Ryan [46].

Primary antibodies used were polyclonal integrin β1 (1∶500–1∶1000; Chemicon), polyclonal integrin β1 against the extracellular domain (M-106, 1∶500; Santa Cruz), monoclonal clathrin heavy chain (clone X22, 1∶1000; AbCam), polyclonal clathrin light chain, consensus sequence (1∶2000; [102]), monoclonal a-adaptin subunit of AP-2 (clone AP6, 1∶1000; AbCam), polyclonal FAK (1∶1000–1∶2000; Upstate), polyclonal ILK (1∶500; Upstate/Chemicon), monoclonal anti- active human integrin β1 (HUTS-21, 1∶500; BD), polyclonal FAK PSSA sampler pack (pY397, pY407, pY576, pY577, pY861, 1∶500–1∶1000; Biosource/Invitrogen), chicken anti-GFP, rabbit anti-HA tag (1∶1000; Santa Cruz), rabbit anti-T7 tag (1∶100; Novagen), GM130-FITC (1∶100, BD), monoclonal anti-a-tubulin (clone DM1A, 1∶1000; Sigma), monoclonal anti-Disabled-2/p96 (clone 52, 1∶500; BD Transduction Labs), polyclonal Numb (1∶250–1∶500; Cell Signaling), polyclonal caveolin-1 (1∶500; Cell Signaling), monoclonal nestin (1∶100; Pharmingen), rhodamine-phalloidin (1∶250; Molecular Probes). Secondary antibodies were used at 1∶500 from Jackson Labs (goat anti-mouse IgG conjugated to Cy2/Cy3/Cy5, goat anti-rabbit Cy2/Cy3) or from Molecular Probes (goat anti-mouse IgG or rabbit IgG conjugated to Alexa 488/594/680, goat anti-chicken Alexa 488).

### Transfection

SVZa explants were transfected as previously described [103]. SVZa explants were dissociated into a single cell suspension using trypsin. Dissociated SVZa cells were then transfected via Amaxa nucleofector with rat nucleofection solution (program G13). Cells were transfected with 1–2 µg of cytoplasmic marker constructs (GFP, tdTomato, or mCherry) to show morphology. Cells transfected with dynamin constructs received 2 µg of dynamin construct and 1 µg of the cytoplasmic marker. Transfected cells were reaggregated in hanging drops, embedded into gel substrate the next morning and allowed to migrate for 4–8 hours before imaging or processing.

### SVZa Explant Culture

The migration substrate was a mixture of phenol red-free, growth factor reduced Matrigel (BD) and rat tail collagen I (BD). Explants were either: 1) mixed directly (1∶1) with the gel substrate and allowed to polymerize at 37°C, 5% CO_2_ for 30–45 min before adding culture media, or 2) added to the top of a gel pad that had polymerized for 10–15 min at 37°C, 5% CO_2_, and allowed to settle into the gel pad. Culture media was composed of phenol red-free Neurobasal supplemented with B27, pen/strep, glutamine, and glucose. Imaging media was culture media with 30 mM HEPES.

Inhibitors (MDC and MiTMAB) were dissolved in DMSO to create 100 mM stock solutions that were diluted in culture or imaging media at the specified concentrations for experiments. For migration distance experiments, explants were continuously cultured in the presence of inhibitors diluted in the media for 4–10 hrs. Inhibitors were always added within 2–3 hrs of embedding, after explants were firmly embedded in the substrate but before most cells exited the explants. For immunostaining experiments, explants were cultured in normal culture media for 4–6 hrs to allow cells to exit the explants, then inhibitors were added for 2–3 hrs prior to histological processing.

### Time-lapse Imaging

Time-lapse imaging was performed using a Zeiss Axiovert 200 M inverted microscope with a heated stage and Orca ER cooled CCD camera (Hamamatsu), or a CoolSnap HQ camera for fluorescence time-lapse. Images were acquired every minute using either OpenLab or Slidebook software. For experiments involving inhibitors, explants were first cultured in imaging media (pre), which was then replaced by imaging media containing the inhibitor at specified concentrations for 30 minutes (during). For washout, explants were rinsed with pre-warmed DMEM that was then replaced by imaging media.

Time-lapse images acquired every minute were used to generate kymographs by an ImageJ plugin written by J. Rietdorf and A. Seitz. The position of the cell rear at the start and end of each period (pre, during, washout) divided by length of time was used to calculate average velocity. Time between translocations was the length of time between the initiation of two discrete movements of the cell rear.

### Electron microscopy

EM images were collected as previously described [5]. Briefly, an isolated neuron with a prominent dilation in its leading process was identified by time-lapse microscopy. As soon as the nucleus moved and/or the rear of the cell contracted, 250 µl of EM grade 8% glutaraldehyde was added to the imaging dish (1 ml total). Samples were processed for EM, embedded in Epon, and sectioned with a Leica Ultracut S ultramicrotome. The imaged cell was located by taking thick sections that were stained on a glass slide with Giemsa stain for orientation. Thin (85-nm) sections were then taken and post-stained with 1∶1 saturated uranyl acetate/acetone for 15 s, then 3 min of 0.3% lead citrate. Samples were rinsed and air-dried. Transmission EM was then performed by using a JEOL 1230 microscope, and images were acquired with a Gatan 967 slow-scan charge-coupled device (CCD) camera.

### Immunohistochemistry

Explants processed for immunocytochemistry were fixed in 2% PFA for 20 min at room temperature. Electroporated brains were fixed in 4% PFA overnight at 4°C, cryoprotected in 30% sucrose in PBS, and embedded in Tissue-Tek O.C.T. (Sakura). Fixed explants or 15 µm thick cryostat sections from electroporated brains were incubated in blocking solution (explants: 15% heat-inactivated goat serum, 50 mg/ml BSA, 0.03% Triton X-100 in PBS; sections: 2% heat-inactivated goat serum, 0.03% Triton X-100, 50 mg/ml BSA in PBS) for 30 minutes–1 hour at room temperature and then incubated with primary antibodies diluted in blocking solution overnight at 4°C. After washing with PBS, samples were incubated in secondary antibody and DAPI diluted in blocking solution for 1.5 hours at room temperature. Experiments examining transferrin, dextran, or surface integrin β1 internalization used blocking solution without Triton X-100. Immunofluorescence images were taken either on a Zeiss LSM510 confocal microscope or a Nikon Eclipse 80*i* epifluorescent microscope.

### Image analysis

Line-scan analysis was performed in ImageJ using the line selection tool to measure fluorescence intensity from cell rear to tip in confocal Z-stack maximum projections. Intensity values along the length of each cell were scaled relative to the maximum intensity value in that cell, then normalized for cytoplasmic volume using the fluorescence intensity of a cytoplasmic marker. Relative intensities were averaged to create 50 binned values along each cell length to compare across cells. The positions of some cells were aligned using the maximal intensity of DAPI nuclear staining. Average intensities of staining at cell rears include values in all bins posterior to the position of maximal DAPI fluorescence, while intensities for cell anteriors include values in all bins anterior to the position of maximal DAPI fluorescence.

For colocalization analysis, optical sections of confocal image Z-stacks were manually divided into 5 regions based on morphology: tip of the leading process (growth cone tip to wrist); distal leading process (wrist to dilation start); dilation (enlarged cytoplasmic region proximal to soma); soma (dilation end to nucleus end); and rear (nucleus end to cytoplasm end). The JACoP ImageJ plugin was used to calculate Pearson's coefficient for each region [104].

Explant migration distance analysis was performed by counting cell nuclei in equally spaced concentric rings beyond the edge of the explant. Cortex migration distances were analyzed by counting GFP+ cells in 10 equally-sized bins from pial surface to ventricle.

### In utero electroporation

In utero electroporation of Swiss/Webster mouse embryos at E13.5 (day of plug = E0.5) was performed as previously described [105]. 0.5 µg/µl GFP with 1 µg/µl of the construct of interest dissolved in PBS containing Fast Green to visualize the injection was injected into the embryonic lateral ventricle with a glass micropipette. Tweezer-type electrode paddles were used to deliver 5×50 ms pulses at 40 V at intervals of 950 ms with an electroporator (ECM830; BTX). Uterine horns were returned to the abdominal cavity to allow embryos to continue developing. Electroporated brains were collected at E15.5, E17.5, or P1 and processed for immunohistochemistry.

### Functional blocking of integrin β1 in SVZa explants

Explants were cultured as above in the presence of anti-integrin β1 (Ha2/5; BD Biosciences) function-blocking antibody or hamster IgM isotype control antibody (G235-1; BD Biosciences) added to the culture at a final concentration of 10 or 50 µg/ml. Explants were cultured for 19–24 hours, then fixed, stained with DAPI and images were captured for migration distance analysis. Migration distance analysis was performed by averaging the cell nucleus position measured at the farthest distance from the edge of the explant at 4 points around the explant.

### Transferrin, dextran, and integrin β1 internalization

To examine transferrin internalization, human transferrin conjugated to Alexa 594 (Invitrogen) was added to the culture medium at 50 µg/ml. To examine bulk membrane uptake, Texas Red conjugated dextran (Invitrogen) was added to the culture medium at 10 µg/ml. To examine surface integrin β1 levels, fixed non-permeabilized cells were immunostained using an antibody against the extracellular domain of integrin β1. For inhibitor experiments, explants were cultured in normal culture media for 6–7 hrs to allow cells to exit explants, then inhibitors were added for 30 min total. After 15 min of exposure to inhibitors, either Alexa 594-transferrin or Texas Red-dextran was added to the media and returned to 37°C, 5% CO_2_ for the remaining 15 min. Explants were then placed on ice, rinsed with cold DMEM, and fixed with cold 2% PFA at room temperature for 20 min. To examine surface integrin β1 levels, explants were exposed to inhibitors for 30 min and then processed for immunohistochemistry.

For transferrin hot-spot analysis, explant dishes were acclimated to room temperature before Alexa 594-transferrin diluted in pre-warmed unsupplemented Neurobasal was added and placed at 37°C, 5%CO_2_ for 10, 5, or 2 min. Explants were then placed on ice, rinsed with cold DMEM, and fixed with cold 2% PFA at room temperature for 20 min.

Internalized puncta were counted using ImageJ by first creating a mask of GFP+ cells to create a masked image of the puncta, and then using the ImageJ analyze particles function to count numbers of internalized puncta.

## Supporting Information

Figure S1 **Clathrin localization to the dilation is specific, and clathrin adaptors colocalize with integrin β1.** (A) Immunostaining for Golgi (GM130) and clathrin light chain. Subtracting the Golgi image from the clathrin image shows that not all clathrin in the dilation is Golgi-associated. (B) Average line scans for caveolin-1 normalized by cytoplasmic GFP (n = 6). (C–E) Migration was inhibited by (D) the presence of 10 µg/ml integrin β1-blocking antibody (Ha2/5) in the media compared to (C) an isotype control (IgM) showing that integrin β1 mediates SVZa neuron migration in a Matrigel/collagen substrate. (F–N) Immunostaining for integrin β1 (red arrows) and clathrin adaptors (green arrows): (F) AP-2, (I) Dab2, and (L) Numb. Average Pearson's coefficients for each subcellular region for (G) AP-2 (n = 12), (J) Dab2 (n = 9), (M) Numb (n = 6). (H, K, N) show significant colocalization for different subcellular regions. R = rear, S = Soma, D = dilation, LP = distal leading process, T = tip. Data presented as mean ± SEM, * p<0.05, ** p<0.01, Student's t-test.
(TIF)

Figure S2 **MiTMAB blocks CME and increases surface integrin β1 levels, but does not block bulk fluid phase uptake.** (A) CME, assayed by transferrin internalization, is significantly inhibited in the presence of 50 and 100 µM MiTMAB. (B) Bulk fluid-phase uptake, assayed by dextran internalization, is not affected until 100 µM MiTMAB. (C) Surface integrin β1 levels were increased in the presence of 30 µM MiTMAB. Data presented as mean ± SEM, * p<0.05, ** p<0.01, Student's t-test.
(TIF)

Figure S3 **MDC blocks CME, but not bulk fluid phase uptake or surface integrin β1 levels.** (A) CME, assayed by transferrin internalization, is significantly inhibited in the presence of 50 and 100 µM MDC. (B) Bulk fluid-phase uptake, assayed by dextran internalization, and (C) surface integrin β1 levels do not appear affected by MDC treatment. Data presented as mean ± SEM, * p<0.05, ** p<0.01, Student's t-test.
(TIF)

Figure S4 **Individual cells show varied adhesion distributions.** Individual and average line scans of fluorescence intensity along the lengths of migrating neurons in control (A–C) and MiTMAB-treated cells (D–F) for (A, D) integrin β1, (B, E) active integrin β1, and (C, F) FAK. Each colored line is the relative fluorescence intensity normalized by cytoplasmic GFP for an individual cell consolidated into 50 bins along the cells' length and aligned by maximal DAPI value. The averaged data for the adhesion molecule and DAPI are represented by thick black and blue lines, respectively.
(TIF)

Figure S5 **GFP+ neurons express dominant negative constructs.** (A–C) Some but not all GFP+ cells at E15 in the IZ express the T7 tag. (D–F) At E17, GFP+ cells at the top of the CP are still positive for T7 tag. (G–L) GFP+ cells at E15 express the dynamin I construct assayed by immunostaining for the HA tag present in the WT-dyn and K44A-dyn constructs. (M–R) At E17, GFP+ cells in the CP of brains electroporated with K44A-dyn are positive for the HA tag (P,Q insets), but GFP+ cells in the IZ express higher levels of HA.
(TIF)

Figure S6 **Radial glia are intact in dominant negative-expressing cortex.** Nestin (red, grayscale) staining shows that radial glial morphology in brains expressing (B, D) DN-Hub or (F, H) K44A-dyn at E15 and E17 look similar to radial glia in brains expressing (A, C) GFP only or (E, G) WT-dyn.
(TIF)

Figure S7 **Dominant negative dynamin-expressing neurons extend axons but their cell bodies are present in the white matter at P1.** Most neurons expressing K44A-dyn fail to migrate into the CP by P1. (A, B, E) The greatest percentage of K44A-dyn-expressing cells is present in the white matter and the distribution of K44A-dyn cells differs significantly from that of WT-dyn cells (p<0.01, Chi-square test). GFP+ cells in the white matter show staining for HA (red), and have the morphology of differentiated neurons (B, inset). (C, D) Both WT-dyn and K44A-dyn expressing neurons extend HA+ axons across the corpus callosum.
(TIF)

Movie S1 **Time-lapse movie of migrating neurons expressing WT-dyn+tdTomato or K44A-dyn+GFP.** Movie of the cells shown in Figure 2C. Images taken every minute.
(MOV)

Movie S2 **Time-lapse movie of a migrating neuron treated with MiTMAB.** Movie of the cell shown in Figure 3C, left. Images taken every minute.
(MOV)

Movie S3 **Time-lapse movie of a migrating neuron treated with MiTMAB.** Movie of the cell shown in Figure 3C, right. Images taken every minute.
(MOV)

Movie S4 **Time-lapse movie of a migrating neuron treated with MDC.** Movie of the cell shown in Figure 4C, left. Images taken every minute.
(MOV)

Movie S5 **Time-lapse movie of a migrating neuron treated with MDC.** Movie of the cell shown in Figure 4C, right. Images taken every minute.
(MOV)
