# Supplementary figures and images for: Endocytosis Regulates Cell Soma Translocation and the Distribution of Adhesion Proteins in Migrating Neurons
Source: PLoS One. 2011 Mar 22;6(3):e17802. doi: 10.1371/journal.pone.0017802 (PMC3062553; doi:10.1371/journal.pone.0017802)

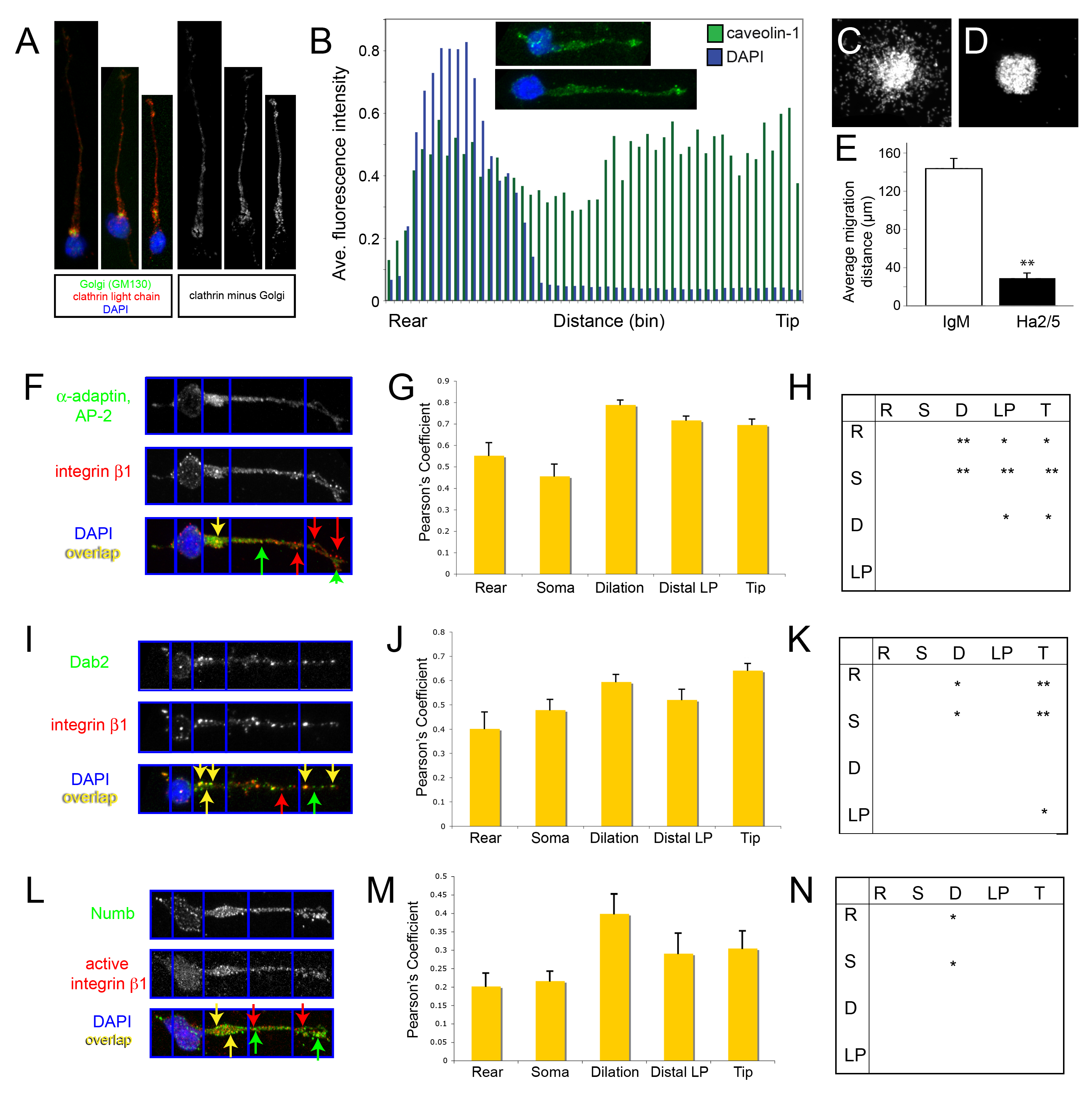

Supplement: Figure S1 — Clathrin localization to the dilation is specific, and clathrin adaptors colocalize with integrin β1. (A) Immunostaining for Golgi (GM130) and clathrin light chain. Subtracting the Golgi image from the clathrin image shows that not all clathrin in the dilation is Golgi-associated. (B) Average line scans for caveolin-1 normalized by cytoplasmic GFP (n = 6). (C–E) Migration was inhibited by (D) the presence of 10 µg/ml integrin β1-blocking antibody (Ha2/5) in the media compared to (C) an isotype control (IgM) showing that integrin β1 mediates SVZa neuron migration in a Matrigel/collagen substrate. (F–N) Immunostaining for integrin β1 (red arrows) and clathrin adaptors (green arrows): (F) AP-2, (I) Dab2, and (L) Numb. Average Pearson's coefficients for each subcellular region for (G) AP-2 (n = 12), (J) Dab2 (n = 9), (M) Numb (n = 6). (H, K, N) show significant colocalization for different subcellular regions. R = rear, S = Soma, D = dilation, LP = distal leading process, T = tip. Data presented as mean ± SEM, * p<0.05, ** p<0.01, Student's t-test. (TIF) [file pone.0017802.s001.tif]

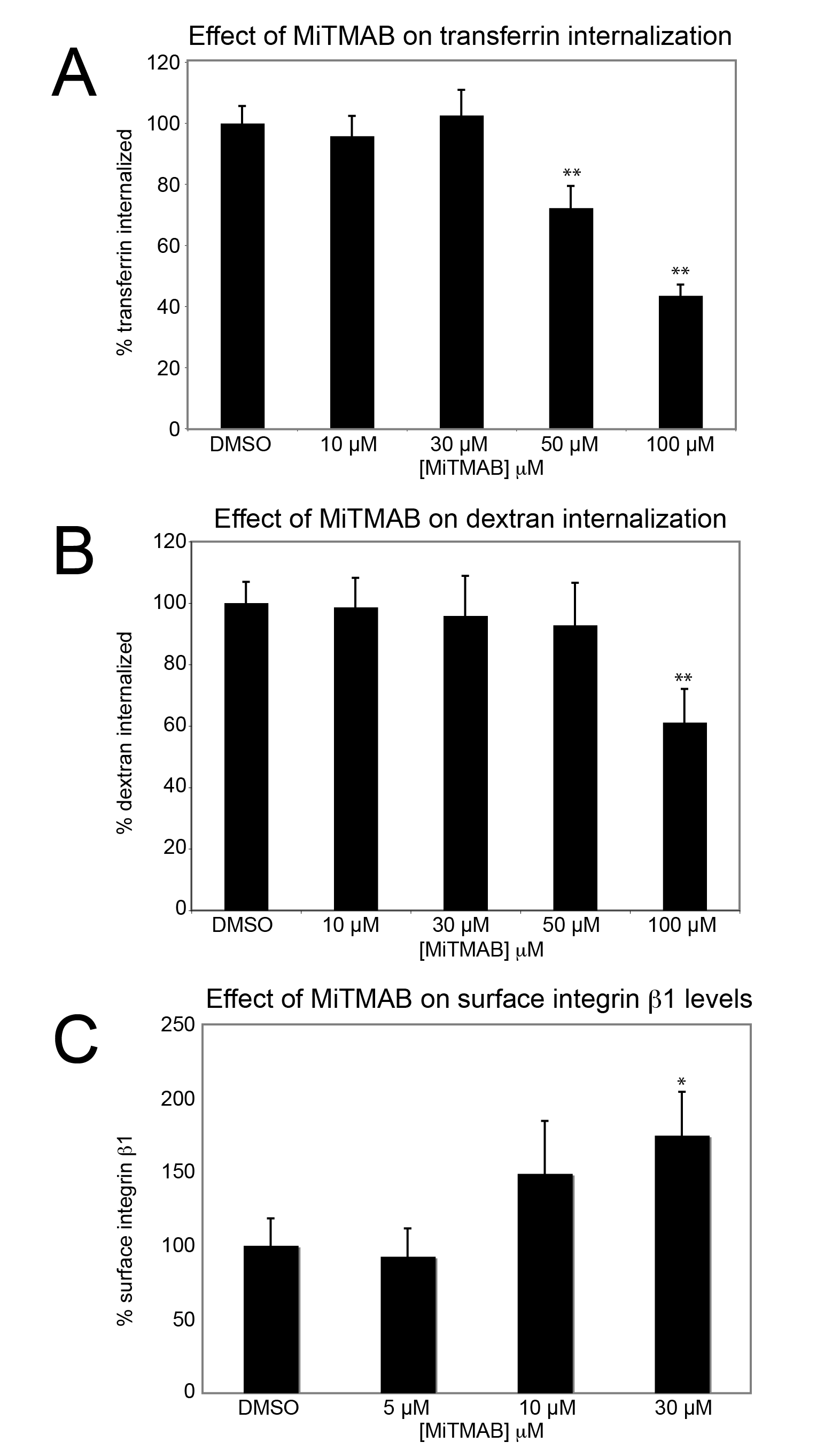

Supplement: Figure S2 — MiTMAB blocks CME and increases surface integrin β1 levels, but does not block bulk fluid phase uptake. (A) CME, assayed by transferrin internalization, is significantly inhibited in the presence of 50 and 100 µM MiTMAB. (B) Bulk fluid-phase uptake, assayed by dextran internalization, is not affected until 100 µM MiTMAB. (C) Surface integrin β1 levels were increased in the presence of 30 µM MiTMAB. Data presented as mean ± SEM, * p<0.05, ** p<0.01, Student's t-test. (TIF) [file pone.0017802.s002.tif]

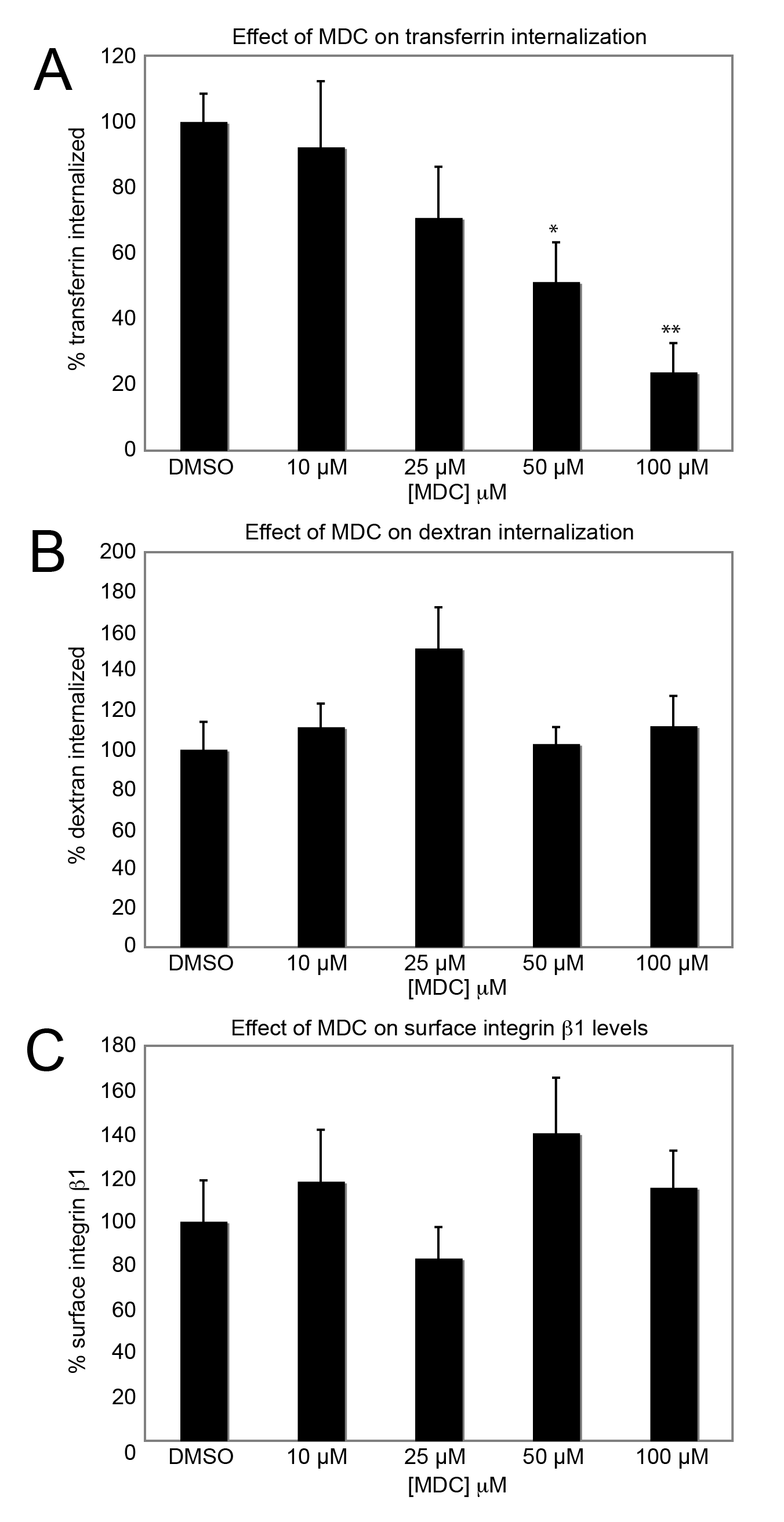

Supplement: Figure S3 — MDC blocks CME, but not bulk fluid phase uptake or surface integrin β1 levels. (A) CME, assayed by transferrin internalization, is significantly inhibited in the presence of 50 and 100 µM MDC. (B) Bulk fluid-phase uptake, assayed by dextran internalization, and (C) surface integrin β1 levels do not appear affected by MDC treatment. Data presented as mean ± SEM, * p<0.05, ** p<0.01, Student's t-test. (TIF) [file pone.0017802.s003.tif]

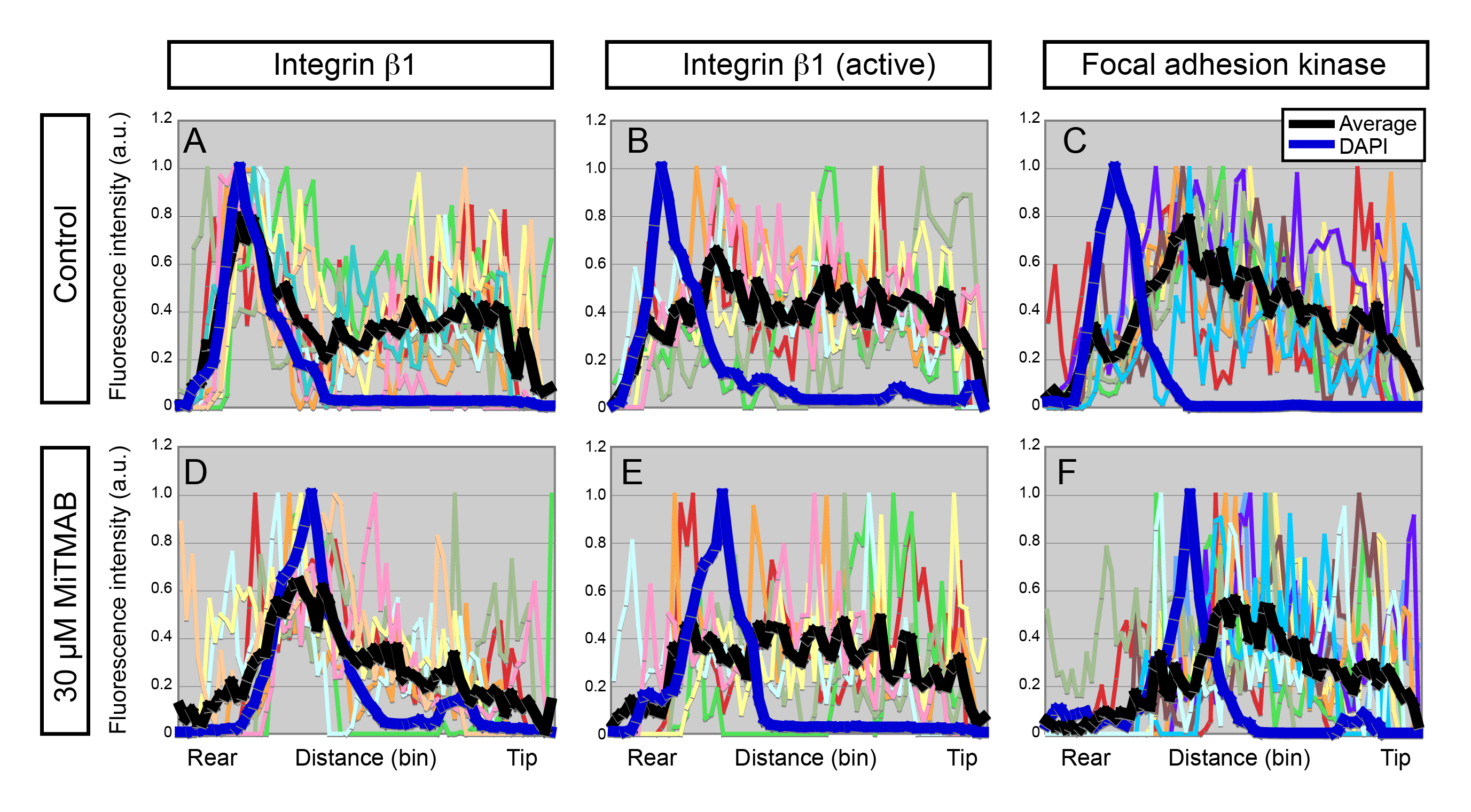

Supplement: Figure S4 — Individual cells show varied adhesion distributions. Individual and average line scans of fluorescence intensity along the lengths of migrating neurons in control (A–C) and MiTMAB-treated cells (D–F) for (A, D) integrin β1, (B, E) active integrin β1, and (C, F) FAK. Each colored line is the relative fluorescence intensity normalized by cytoplasmic GFP for an individual cell consolidated into 50 bins along the cells' length and aligned by maximal DAPI value. The averaged data for the adhesion molecule and DAPI are represented by thick black and blue lines, respectively. (TIF) [file pone.0017802.s004.tif]

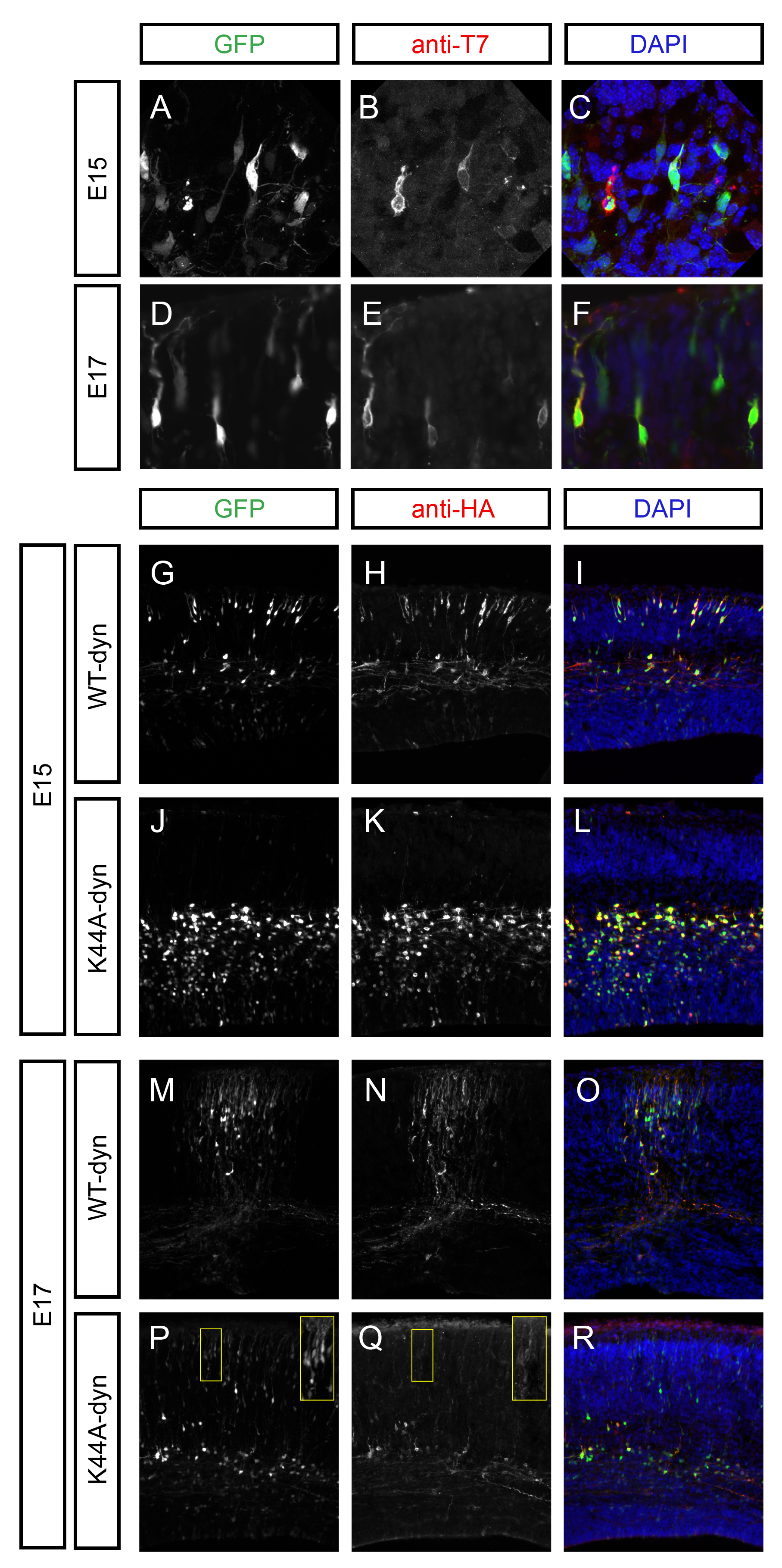

Supplement: Figure S5 — GFP+ neurons express dominant negative constructs. (A–C) Some but not all GFP+ cells at E15 in the IZ express the T7 tag. (D–F) At E17, GFP+ cells at the top of the CP are still positive for T7 tag. (G–L) GFP+ cells at E15 express the dynamin I construct assayed by immunostaining for the HA tag present in the WT-dyn and K44A-dyn constructs. (M–R) At E17, GFP+ cells in the CP of brains electroporated with K44A-dyn are positive for the HA tag (P,Q insets), but GFP+ cells in the IZ express higher levels of HA. (TIF) [file pone.0017802.s005.tif]

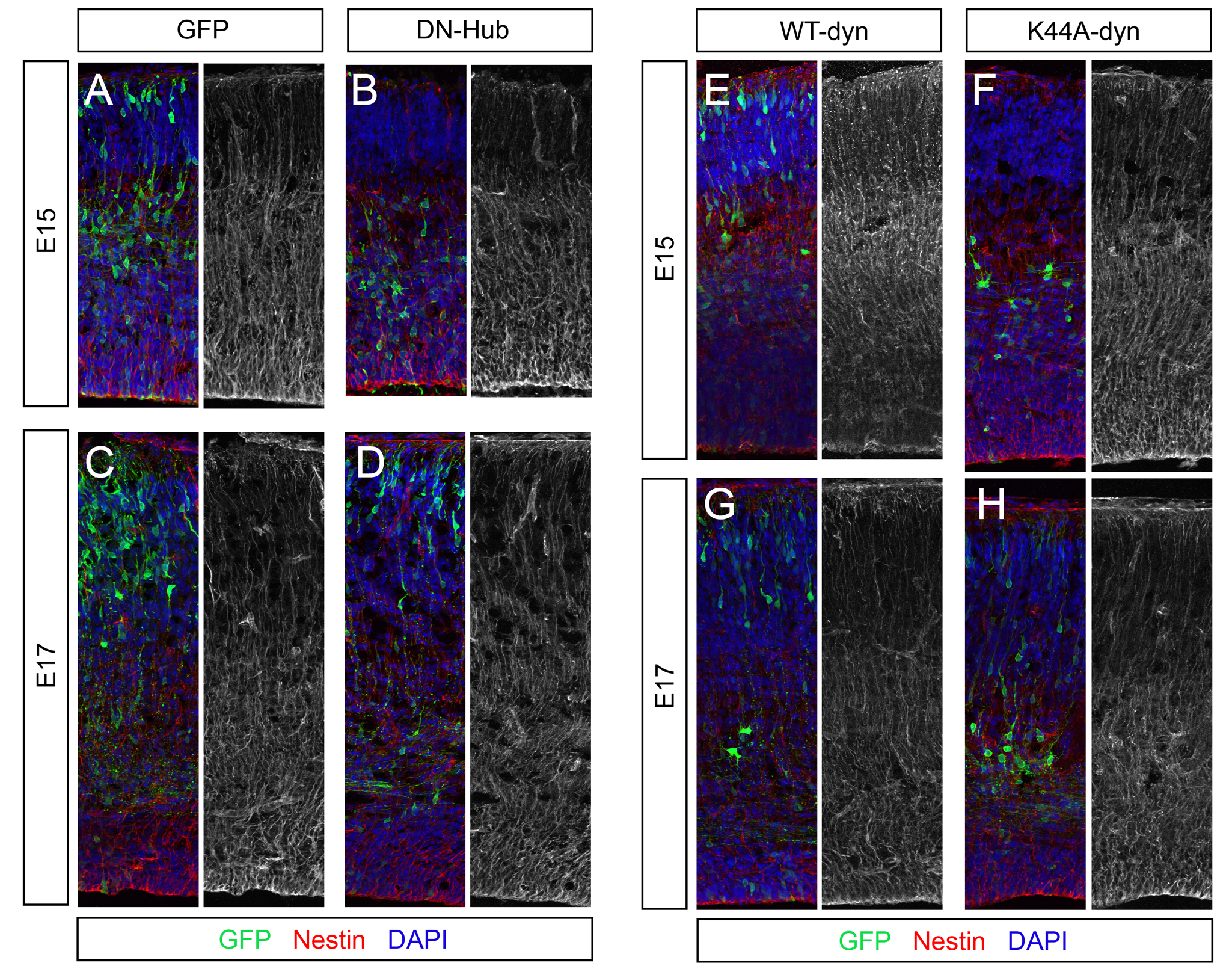

Supplement: Figure S6 — Radial glia are intact in dominant negative-expressing cortex. Nestin (red, grayscale) staining shows that radial glial morphology in brains expressing (B, D) DN-Hub or (F, H) K44A-dyn at E15 and E17 look similar to radial glia in brains expressing (A, C) GFP only or (E, G) WT-dyn. (TIF) [file pone.0017802.s006.tif]

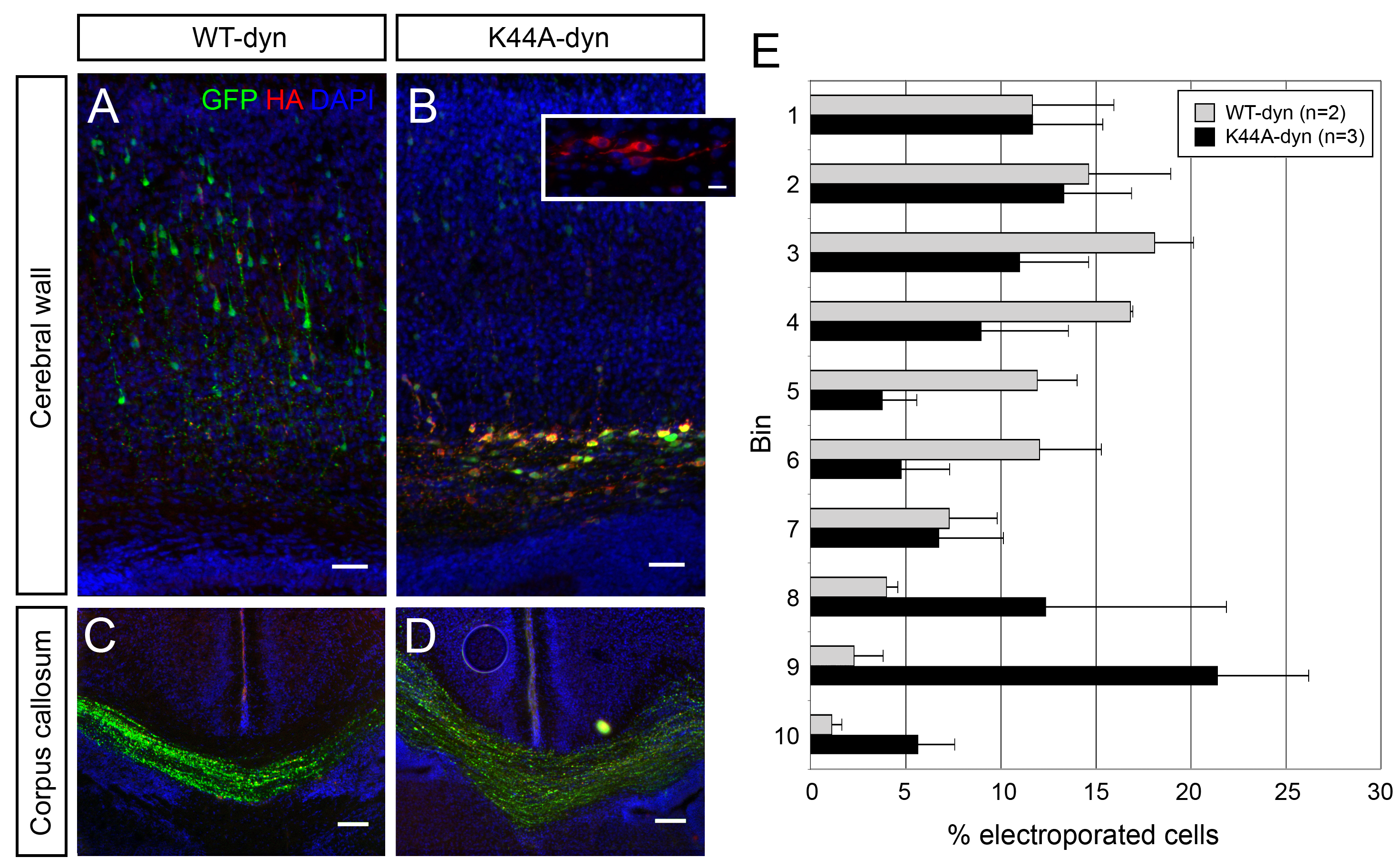

Supplement: Figure S7 — Dominant negative dynamin-expressing neurons extend axons but their cell bodies are present in the white matter at P1. Most neurons expressing K44A-dyn fail to migrate into the CP by P1. (A, B, E) The greatest percentage of K44A-dyn-expressing cells is present in the white matter and the distribution of K44A-dyn cells differs significantly from that of WT-dyn cells (p<0.01, Chi-square test). GFP+ cells in the white matter show staining for HA (red), and have the morphology of differentiated neurons (B, inset). (C, D) Both WT-dyn and K44A-dyn expressing neurons extend HA+ axons across the corpus callosum. (TIF) [file pone.0017802.s007.tif]
